# The Role of Cognitive Control in Older Adult Cognitive Reappraisal: Detached and Positive Reappraisal

**DOI:** 10.3389/fnbeh.2017.00027

**Published:** 2017-03-07

**Authors:** Ying Liang, Meng Huo, Robert Kennison, Renlai Zhou

**Affiliations:** ^1^Department of Psychology, School of Social and Behavioral Sciences, Nanjing UniversityNanjing, China; ^2^Department of Social Work and Social Policy, Nanjing UniversityNanjing, China; ^3^Department of Human Development and Family Sciences, The University of Texas at AustinAustin, TX, USA; ^4^Department of Psychology, California State University, Los AngelesLos Angeles, CA, USA

**Keywords:** cognitive control, cognitive reappraisal, detached and positive reappraisal, aging

## Abstract

Older adults are more likely to regulate their emotions by engaging in cognitive reappraisal. However, depending on the type of cognitive reappraisal used, efforts to regulate emotions are sometimes met with success and other times with failure. It has been suggested the well-known age-related decline in cognitive control might be the culprit behind the poor use of detached reappraisal by older adults. However, this possibility has not been thoroughly investigated. In addition, studies have not examined what aspects of cognitive control– shifting, updating or inhibition–might be relevant to cognitive reappraisal. In the present study, 41 older participants were tested on cognitive control and abilities to use detached and positive reappraisal. Results showed detached reappraisal compared to positive relied more heavily on cognitive control, specifically mental set shifting. Results of this study have important implications for development of cognitive training interventions for older adults.

## Introduction

Emotion regulation is an essential element of human psychological well-being (Schmeichel et al., 2008). Previous studies (Gross and John, 2003; John and Gross, 2004) have developed many types of emotion regulation strategies, e.g., situation selection, situation modification, attentional deployment, cognitive reappraisal, and behavioral suppression. Among various strategies, cognitive reappraisal, which refers to reinterpreting an emotional situation in order to change physiological and emotional responses (Gross, 1998; Urry and Gross, 2010), has been most commonly used and studied. Compared to younger adults, older adults are more likely to regulate their emotions by engaging in cognitive reappraisal (John and Gross, 2004; Urry and Gross, 2010). However, two subtypes of reappraisal, detached and positive reappraisal, have different age-related trajectories with regards to success rate; the use of detached reappraisal declines with age whereas the use of positive reappraisal increases with age (Shiota and Levenson, 2009). Shiota and Levenson (2009) argued that differential reliance on cognitive control may explain these different trajectories, but this possibility has not been fully investigated. Based on related research, no study to date has examined what aspects of cognitive control, i.e., shifting, updating and inhibition–are responsible for the differential reliance of different cognitive reappraisals. This study examined specific aspects of cognitive control in older adults and their ability to use detached and positive reappraisal when exposed to emotional stimuli.

### Cognitive Reappraisal in Older Adults

In spite of cognitive and physical declines, older adults tend to enjoy positive emotional lives (Carstensen and Mikels, 2005). Both cross-sectional (Stawski et al., 2008) and longitudinal (Charles et al., 2001; Cacioppo et al., 2008) studies have demonstrated older adults are generally happier than their younger counterparts, reporting higher levels of positive affect and lower levels of negative affect compared to other age groups. Older adults are strongly influenced by their perception of a limited time horizon and thus, have a tendency to emphasize emotion regulation (Carstensen, 1992, 1995).

One of the widely used and most-studied regulatory strategies in older adults is cognitive reappraisal, which has appeared to be an effective strategy in every-day life (Giuliani and Gross, 2009; McRae et al., 2012a). Previous researches have shown cognitive reappraisal leads to decreased expressions of negative emotions and their behaviors (Gross, 1998), decreased startle responses (Dillon and LaBar, 2005) and attenuated autonomic responses (Gross, 1998). Cognitive reappraisal includes two subtypes, detached reappraisal and positive reappraisal (Schmeichel et al., 2008; Shiota and Levenson, 2009; Urry and Gross, 2010; McRae et al., 2012b). In detached reappraisal, individuals deliberately consider the situation from an unemotional and detached perspective; whereas in positive reappraisal, individuals focus on negative aspects of the situation and try to reinterpret the situation in terms of potential positive outcomes. Multiple studies have demonstrated that older adults are likely to use cognitive reappraisal more frequently than younger adults (Urry and Gross, 2010; Opitz et al., 2012). However, these two subtypes of reappraisal, detached and positive reappraisal, have different age-related trajectories with regards to success rate. Sampling 144 adults residing in the Northern California Bay Area, Shiota and Levenson (2009) found that older adults were less successful at using detached reappraisal than younger adults, but are more successful at using positive reappraisal. They argued that differential reliance on cognitive control may explain these varying trajectories. But this has not been fully investigated. Therefore, this research further examines the relationship between cognitive reappraisal and cognitive control.

### Cognitive Control and Cognitive Reappraisal

Like any complex activity requiring cognitive control, successful cognitive reappraisal also relies on cognitive control (Ochsner and Gross, 2005, 2007, 2008; Schmeichel et al., 2008; Opitz et al., 2012), which represents selection, planning, coordination, and execution of goal-driven thoughts and actions (Miller and Cohen, 2001). This thinking is propagated by the common interpretation that neural regions engaged in cognitive reappraisal partially overlap with those more broadly involved in cognitive control (Ochsner and Gross, 2005; Kalisch, 2009). Previous studies have demonstrated there are many types of cognitive control processes. As unity/diversity framework (Miyake et al., 2000; Friedman et al., 2006; Miyake and Friedman, 2012) described, cognitive control is made up of at least three correlated, yet distinct sub-processes: mental set shifting, information updating and monitoring, and inhibition of prepotent responses, which are most commonly categorized as (McRae et al., 2012b).

Older adults have shown impaired cognitive control abilities (Salthouse, 2004, 2009, 2011). The two subtypes of cognitive reappraisal do not appear to be equally affected by age and may not rely on cognitive control mechanisms to the same degree (John and Gross, 2004; Shiota and Levenson, 2009; Opitz et al., 2012). It has been suggested that this may account for the disparate success rate of different cognitive reappraisal strategies (Shiota and Levenson, 2009; Urry and Gross, 2010; Opitz et al., 2012). Specifically, in detached reappraisal, shifting from an emotional perspective to a detached perspective is thought to draw heavily on cognitive control (Shiota and Levenson, 2009). Indeed, Shiota and Levenson (2009) found age-related linear decline in the use of detached reappraisal. As positive reappraisal requires individuals to maintain focus on emotion rather than ignore it, it is less likely to rely on cognitive control compared to detached reappraisal. Consistent with this notion, studies on positive reappraisal have not revealed an associated age-related decline (Shiota and Levenson, 2009).

Several important questions remain to be answered in order to gain a more complete understanding of the role of cognitive control in older adult cognitive reappraisal. Firstly, few studies have examined the relationship between cognitive reappraisal and cognitive control. According to related research, there is only one empirical investigation of reappraisal ability have included the specific aspects of cognitive control (McRae et al., 2012a). This study measured cognitive control from set shifting, response inhibition, and working memory updating, revealing a positive relationship between reappraisal frequency and cognitive control. However, this research was not able to assess cognitive control mechanisms underlying each subtype of cognitive reappraisal. Furthermore, different from McRae’s study, this paper made the participant groups accurate to the old adults. Secondly, most previous studies assumed age-related declines in cognitive control were responsible for inferior implementation of detached reappraisal in older adults (Schmeichel et al., 2008; Shiota and Levenson, 2009; Urry and Gross, 2010; Opitz et al., 2012); however, such studies did not involve all of the sub-processes likely to sub-serve cognitive control. Therefore, it remains unclear what specific cognitive control ability might explain the larger reliance of detached reappraisal.

### The Present Study

The present study sought to examine specific aspects of cognitive control– shifting, updating, inhibition– in older adults as well as their ability to use each type of reappraisal when exposed to emotional stimuli. This work followed a recent trend in emotion regulation and coping research, which recognizes that differential reliance on cognitive control may lead to two subtypes of reappraisal, detached and positive reappraisal, which have different age-related trajectories with regards to success rate (Shiota and Levenson, 2009). This study added to the prior study by exploring and assessing specific cognitive control mechanisms underlying each subtype of cognitive reappraisal in older adults. Furthermore, as no study examined what aspects of cognitive control– shifting, updating and inhibition– are responsible for differential reliance of different cognitive reappraisals. This study aimed to provide preliminary analyses to examine the relationship between them. Hypotheses were as follows:

Hypothesis 1: We hypothesize that participants’ changes in self-reported emotional experience and physiological reactions in detached reappraisal have a heavier reliance on cognitive control than in positive reappraisal.

Hypothesis 2: We expect that participants’ detached-reappraisal-related changes in subjective feelings and Heart Rate (HR) are more highly correlated with mental set shifting than other cognitive control abilities.

## Materials and Methods

### Participants

The study was approved by the Institutional Review Board of School of Psychology at Beijing Normal University, and all participants gave written informed consent and were paid for their participation. Forty-one older adults between ages 55–72 years [23 women, *M*_age_ = 61.8 years, *SD*_age_ = 4.66, 18 men, *M*_age_ = 63.5 years, *SD*_age_ = 4.53, no significant age difference between gender groups was found, *t*(39) = -1.19, *p* > 0.05], with the diploma of senior middle school or above [23 women, *M*_edu_ = 14.26 years, *SD*_edu_ = 3.14, 18 men, *M*_edu_ = 13.78 years, *SD*_edu_ = 3.56, no significant school years difference between gender groups was found, *t*(39) = -0.461, *p* > 0.05], were recruited via community postings, word-of-mouth, and through the University of the Third Age (an affiliate of Beijing Normal University). All participants were right-handed, and none reported any history of psychiatric disorders and dementias. All participants were encouraged to wear corrective lenses if needed, and thus all participants had normal or correcting to normal vision. Participants were also screened for cognitive impairment by a minimum score of 30 on the telephone interview gaging cognitive status (Brandt et al., 1988). Participation involved two sessions with a 1 week interval to avoid inferences with each other between the two reappraisal blocks and offer the participants a short- break to return to their baselines. Participants each completed two scales, three cognitive control tests and two emotion regulation tasks.

### Materials

#### Cognitive Control Tests

This study included three measures of cognitive control: the Plus–Minus Test (Spector and Biederman, 1976), the Number Memory Test (Morris and Jones, 1990) and the Magnitude-Size Stroop Test (Stroop, 1935; Wang et al., 2009). All three tests were originally created or adapted for older adults. The Plus–Minus Test was developed to measure the speed and accuracy of individuals shifting from one rule to another. This test consisted of three sub-tests in which three lists of 20 two-digit numbers were presented. In the first sub-test, participants were instructed to add 3 to each number and verbally report answers. In the second sub-test, the rule was changed such that participants subtracted 3 from each number. In the final sub-test, participants were asked to shift between adding 3 to and subtracting 3 from each number. A difference in reaction times (namely shift cost) was computed by subtracting the average reaction time in the first two sub-tests from the reaction time in the final sub-test. The Number Memory Test was developed to measure performance in updating incoming information. This test included number strings, each with certain numbers, presented serially for 1500 ms per number. The length of number strings randomly varied between 7, 9, and 11 digits between trials. Participants were asked to enter the last three numbers of each number string. The number of correct trials was recorded. The Magnitude-Size Stroop Test was developed to measure how efficiently individuals inhibit their prepotent and automatic responses to number size. Three different sets of number pairs were used: the first set had numbers of the same size; the second set had numbers sized in proportion to the magnitude of the number (larger values were larger in size); and the third set included numbers of different sizes whereby number size was inversely proportional to magnitude. Participants were asked to compare the two numbers presented on screen and respond to the number with larger magnitude regardless of size. The difference in reaction times was calculated by subtracting reaction times for the first set from the average reaction times for the second and third set.

#### Emotion Regulation Tasks

The emotion regulation task was a modified version of previously reported emotion regulation paradigms measuring cognitive reappraisal (Gross and John, 2003). This task measures reappraisal ability by computing each participants’ the average magnitude of reduction in negative affect, in response to matched negative images during an instruction to reappraise compared with an instruction to respond naturally. Participants were asked to view 90 colored pictures (800 pixels × 600 pixels)selected from the International Affective Pictures System (IAPS) (Lang et al., 1999). Sixty pictures were selected to be high in arousal (*M* = 6.49, *SE* = 1.95 on a 1–9 scale where 9 signifies *extremely aroused*) and negative (*M* = 7.16, *SE* = 1.54 on a 1–9 scale where 9 signifies *extremely unpleasant*) (Urry, 2010) based on norms for older adults. The remaining 30 pictures were selected to be low in arousal (*M* = 4.12, *SE* = 1.84) and neutral (*M* = 4.16, *SE* = 1.54) (Urry, 2010). Stimuli were presented via E-prime (ver. 1.1) on an IBM-compatible computer with a 14″ screen.

Participants were trained to follow three different kinds of instructions: (a) watching without engaging in emotion regulation (JW block); (b) watching with detached reappraisal (DR block); and (c) watching with positive reappraisal (PR block). The JW block embraced 15 neutral pictures and 15 negative pictures whereas the DR block and PR block each 15 negative pictures. No neutral pictures were included in reappraisal blocks as there was no need to reappraise them. During the process, the three instructions were explained and participants were asked to try reappraisals in several sample pictures before beginning experimental trials. For the DR block, participants were asked to ‘adopt a detached and unemotional attitude, seeing pictures in such a way that they feel less negative emotion.’ For the PR block, participants were instructed to ‘think about positive aspects of what they are seeing, seeing pictures in such a way that they feel less negative emotion’ (Shiota and Levenson, 2009).

Each emotion regulation task consisted of two blocks: one JW block and either the PR or DR block. The JW block was designed to come first followed by a reappraisal block, as watching negative pictures first may change subsequent participant mental status when watching neutral pictures later, consequently making reactions to neutral pictures incomparable under different circumstances. To control for order effects of PR and DR blocks, half the participants were randomly assigned to complete one JW block then one DR block, followed by one JW block and then one PR block 1 week later. The other half were randomly assigned to complete a schedule with order of the two types of reappraisals reversed, completing the PR block first then DR block 1 week later. During these tasks, older adults may be very likely to automatically engage in attentional deployment to positive information rather than to negative (Urry, 2010). To investigate the effects of cognitive reappraisal only, all trials were conducted with gaze direction (Urry, 2010) to hold attentional deployment constant during the process of cognitive reappraisal.

During reappraisal tasks, each picture was presented for 10 s while the participant’s gaze was directed to a specific Area of Interest (AOI) after 6 s. To accomplish this, all parts of the picture with the expection of one square (250 pixels × 250 pixels), the AOI faded out. AOIs were chosen based on ratings collected from independent professional coders. For unpleasant pictures, AOIs were highly arousing; for neutral pictures, AOIs were central. Urry (2010) compared gaze directed to highly arousing areas and non-arousing areas of negative pictures finding emotion regulation effects were only robust in the former condition.

### Measures

#### Subjective Emotional Experience

At the end of each trial, participants were asked to rate their subjective feelings on a 1–9 scale, where 1 is *extremely pleasant and positive*, 5 is *neutral* and 9 is *extremely unpleasant and negative*. For the first block, participants rated their first-sight feelings without regulating emotion. Importantly, for the second block, participants rated their regulated feelings. The average subjective ratings of negative pictures in reappraisal blocks were subtracted from those in the JW blocks and calculated as *D*.

#### Physiological Reaction

Physiological data collection was recorded by a desktop computer running AcqKnowledge software (ver. 4.0) and with a Biopac MP150 bioamplifier (Goleta, CA, USA). Physiological reactions were acquired continuously during the entire reappraisal task and markers separated each picture. As previous study did (Gross, 1998), we chose HR and electrical skin conductance as the physiological reaction indices.

Electrocardiography (ECG) was used to measure HR, which is innervated by the sympathetic and parasympathetic nervous system (Lang and Bradley, 2010; Urry, 2010). During tasks involving viewings of negative pictures, HR is usually associated with initial heart deceleration (Urry, 2010). Three disposable Ag/AgCl electrodes pre-gelled with chloride gel (1 cm circular contact area) were placed on the left wrist and both ankles. ECG was continuously recorded at 500 Hz and bandpass-filtered from 0.5 to 35 Hz.

Electrodermal Activity (EDA) was used to examine the electrical skin conductance, which is primarily controlled by the sympathetic nervous system. EDA has long been used as a measure of emotional responses (Jones, 1935; Schlosberg, 1954; Cooper and Siegel, 1956; Sharma and Kapoor, 2013; Bach, 2014). Two disposable Ag/AgCl electrodes were attached to the distal phalanges of the left index (+) and middle (-) fingers.

An emotion manipulation check was employed in which the average physiological reactions (HR and EDA) to neutral pictures were subtracted from negative pictures in the JW blocks. Reappraisal-related changes were computed by averaging the reactions to DR/PR, and negative pictures were subtracted from the JW negative, calculated as *D*.

### Procedure

Participants were randomly assigned to a morning (8:00 am–12:00 pm), an afternoon (13:30–17:00 pm) or an evening (18:30–22:00 pm) testing session. Upon arrival at the laboratory, participants reviewed and signed a consent form. A research assistant then guided participants to complete the Positive and Negative Affect Schedule (PANAS) (Crawford and Henry, 2004), which is a brief self-reported scale of both positive and negative affect, utilizing a 5-point scale where *1 signifies none or little* and *5 very much*. For each scale, participants reported the extent they felt certain states over the past few weeks on 10 items, including “enthusiastic” and “proud,” for the positive scale and “hostile” or “irritable,’, for the negative scale. The Beck Depression Inventory (BDI) (Beck et al., 1961) was administered along with a self-reported depression scale, in which participants described their levels of a series of depression symptoms, e.g., to what degree do they felt pessimistic about the future.

Subsequently, participants completed a cognitive control test and emotion regulation task. They were assigned to an experimental condition, systematically crossing age, sex, ethnicity, reappraisal type, and stimulus tape. To avoid fatigue shown in the pilot section, participants completed two of the three cognitive control tasks (the Plus–Minus Task and the Magnitude-Size Stroop Test) on the 1st day. The third task (the Number Memory Task) was administered 1 week later. Participants were outfitted with the HR and EDA monitors when they completing one emotion regulation task.

### Analysis

Participant reactions for all three cognitive control conditions were converted into standardized scores. A composite score was used to determine an overall level of cognitive control (Mather and Knight, 2005). Although the three cognitive aspects are distinct, participants who scored high on all three tasks and who had a high composite score were classified as having better cognitive control.

Cognitive reappraisal data consisted of ratings of change in subjective emotional response and physiological reaction. Notably, physiological reactions were only considered for detached reappraisal as positive reappraisal might involve positive emotions after regulation, thus making it difficult to explain changes; if so, physiological reactions could result from a failure of positive reappraisal or an elevation of positive emotions. Subsequently, subjective ratings between the two types of cognitive reappraisals were compared.

The present study employed the independent group *t*-tests, paired group *t*-tests, Pearson correlation and linear regression. Statistical significance was set at *p* < 0.05 level (2-tailed).

## Results

### Demographics and Behavioral Data

For PANAS, participants reported higher positive affect (*M* = 29.12, *SD* = 7.15) than negative affect (*M* = 16.73, *SD* = 6.14). For BDI, participants did not display severe clinical depression symptoms (*M* = 7.37, *SD* = 6.71). Participant self-reports on negative effects were correlated with those on depression symptoms (*r* = 0.36, *p* = 0.02).

For cognitive control tests, scores on the plus–minus test (*M* = 564.49 ms, *SE* = 417.43 ms), the number memory test (*M* = 20.20, *SE* = 5.75), and the magnitude-size test (*M* = 125.56 ms, *SE* = 1.47 ms) were converted to *Z* scores. Note that the plus–minus and the Stroop scores were multiplied by -1 so that higher score was associated with better performance. As shown in **Table 1**, none of these scores were significantly correlated with one another.

**Table 1:** Correlations among cognitive control tasks.

Score	Plus–minus	Number memory	Magnitude-size Stroop
Plus–minus	–		
Number memory	0.19	–	
Magnitude-size Stroop	0.22	0.04	–

*The plus–minus task was used to measure mental set shifting. The number memory task was used to measure information updating. The magnitude-size Stroop task was used to measure inhibition of prepotent responses. The scores on the plus–minus task and the magnitude-size Stroop task were multiplied by –1 so that r–values reflected a correct direction*.

### Emotion Regulation

#### Manipulation Check

In the JW block followed by the DR block, subjective ratings for negative pictures (*M* = 6.62, *SE* = 0.93) were significantly higher than those for neutral pictures (*M* = 4.48, *SE* = 0.96), *t*(40) = 9.86, *p* < 0.001, *d* = 3.12. In the JW block followed by the PR block, subjective ratings for negative pictures (*M* = 6.69, *SE* = 0.87) were higher than those for neutral pictures (*M* = 3.94, *SE* = 1.08), *t*(40) = 12.10, *p* < 0.001, *d* = 3.83. The two JW blocks were compared and no significant difference was found, *t*(39) = 0.48, *p* = 0.63.

The HR data of four participants was excluded due to participants’ leg shaking. Negative pictures produced a significant deceleration in HR (*M* = 113.57, *SE* = 38.39) compared to neutral pictures (*M* = 114.89, *SE* = 38.35) in the JW blocks, *t*(36) = -2.11, *p* = 0.04, *d* = -0.70. For the EDA measure, the data of 10 participants was excluded due to participants’ leg shaking or missing observations. EDA differences were not observed between negative pictures (*M* = 5.92, *SE* = 3.67) and neutral pictures (*M* = 5.90, *SE* = 3.60), *t*(30) = -0.75, *p* = 0.45. Taken together, however, these results indicated that emotional elicitation of the chosen negative pictures was obtained (Urry, 2010).

#### Reappraisal Effects

The effects of cognitive reappraisal were assessed via participants’ changes in subjective ratings of emotional experience and physiological reactions for negative pictures between the JW block and the DR/PR block. For detached reappraisal, a significant decrease in subjective ratings was found, *t*(40) = 7.71, *p* < 0.001, *d* = 2.44, indicating a strong impact of detached reappraisal on emotional experience. Participants also displayed a significant decrease in both HR [*t*(36) = 2.34, *p* = 0.03, *d* = 0.78] and EDA [*t*(30) = 4.25, *p* < 0.001, *d* = 1.55] respectively. Importantly, HR was correlated with subjective experience, *r* = 0.35, *p* = 0.03.

For positive reappraisal, a significant decrease in negative affect was also observed, *t*(40) = 9.00, *p* < 0.001, *d* = 2.85. In addition, positive reappraisal compared to detached reappraisal decreased unpleasant emotions more effectively: the average *d* in the positive reappraisal condition (*M* = 1.98, *SE* = 1.41) was higher than that in the detached reappraisal condition (*M* = 0.78, *SE* = 0.65), *t*(40) = 5.22, *p* < 0.001, *d* = 1.65.

### Hypotheses Testing

**FIGURE 1:**
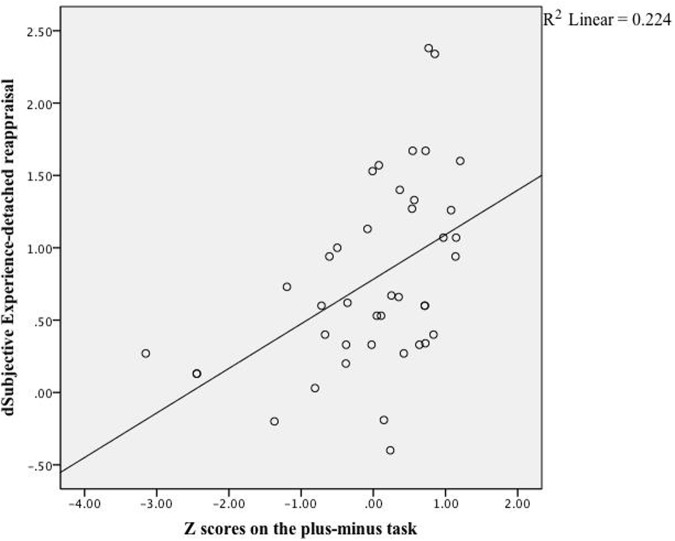
**Linear regression.** The correlation between scores on the plus–minus task and effects (subjective ratings) of detached reappraisal fit the linear equation shown above. Each dot represented data collected from a specific participant.

**FIGURE 2:**
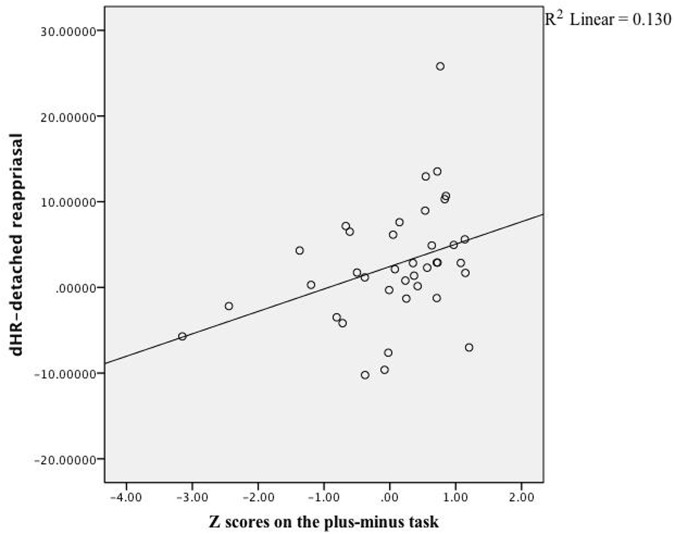
**Linear regression.** The correlation between scores on the plus–minus task and effects (HR) of detached reappraisal fit the linear equation shown above. Each dot represented data collected from a specific participant.

As expected, DR-related changes on subjective feelings were correlated with participants’ scores of cognitive control, *r*(40) = 0.32, *z* = 0.84, *p* = 0.04, whereas PR-related changes were not, *r*(40) = -0.09, *z* = 0.90, *p* = 0.37. Looking into effects of specific cognitive control aspects, only the scores on the plus–minus task, which measured mental set shifting, was strongly correlated with changes in emotional experience (see **Table 2**), *r*(40) = 0.47, *z* = 5.26, *p* < 0.001, see **Figure 1**. In terms of physiological reactions, there was a significant correlation between mental set shifting and HR, *r*(37) = 0.36, *z* = 2.24, *p* = 0.025, see **Figure 2**.

**Table 2:** Cognitive control and cognitive reappraisal.

Score∖d	Detached reappraisal	Positive reappraisal
Plus–minus	0.47^∗∗^	-0.02
Number memory	0.02	0.01
Magnitude-size Stroop	0.15	0.17

^∗^*p < 0.05, ^∗∗^ p < 0.01, ^∗∗∗^ p < 0.001*.

The age-related decline in the success of detached reappraisal has been assumed to have something to do with the age-related decline in cognitive control. Interestingly, however, within such a narrow range of age, participants’ DR-related changes in the composite measure of emotional reactions (0.08 ± 1.72) were not significantly correlated with age, *r*(40) = 0.04, *p* > 0.05. Furthermore, after controlling for age, the composite measure of cognitive control was not correlated to the composite measure of participants’ DR-related changes, *r*(26) = 0.19, *p* = 0.33. However, mental set shifting was observed to still be strongly correlated with participants’ DR-related changes even when age was included as covariate, *r*(26) = 0.39, *p* = 0.04. More specific, the correlations were only found in subjective ratings and HR, *r*(26) = 0.42, *p* = 0.03; HR, *r*(26) = 0.44, *p* = 0.02. This finding suggests that while detached reappraisal is associated with mental set shifting in older adults, this association seems to be present irrespective of age.

To test the first hypothesis of a heavier reliance of detached reappraisal on cognitive control, changes in emotional experience were regressed on the composite score each of the three cognitive control tests. Age, gender, positive and negative effects, depression, and composite scores of each cognitive control test were entered, no evidence of significant prediction of composite score of cognitive control was found [*B* = 0.30, *t*(40) = 1.84, *p* > 0.05].

For specific aspects of cognitive control, a strong prediction of mental set shifting was expected, rather than the other two cognitive control abilities for detached-reappraisal-related effects. The expectation was confirmed in regression models (illustrated in **Table 3**). These models included mental set shifting as a predictor, detached-reappraisal-related changes in subjective feelings, HR and EDA as respective outcomes, and age, gender, effects, depression, information updating and inhibition of prepotent responses as control variables. As expected, mental set shifting was found to predict detached-reappraisal-related changes in subjective feelings and HR. Results showed no association between mental set shifting and effects on EDA [*B* = 0.23, *t*(30) = 1.00, *p* > 0.05].

**Table 3:** Models for detached-reappraisal-related subjective and physiological reactions.

	Subjective ratings^a^		Heart rate^a^	
	*B*	*SE*	*B*	*SE*
Intercept	-0.92	1.40	2.69	2.42
Predictors				
Mental set shifting	0.30^∗∗^	0.11	0.42^∗^	0.19
Information updating	-0.04	0.10	-0.12	0.18
Inhibition of prepotent responses	0.03	0.10	0.09	0.18
Control variables				
Age	0.02	0.02	-0.05	0.04
Gender^b^	0.13	0.22	0.16	0.36
Positive affect	0.02	0.01	0.01	0.02
Negative affect	-0.01	0.02	-0.02	0.03
Depression	0.01	0.02	0.03	0.03

^a^*The outcome was detached-reappraisal-related changes. ^b^0 = male 1 = female.*

^∗^*p < 0.05, ^∗∗^p < 0.01, ^∗∗∗^p < 0.001.*

*For positive reappraisal, we found no evidence of significant relations between positive reappraisal and any factors in the model as expected.*

## Discussion

The present study focused on assessing the specific cognitive control mechanisms underlying each subtype of cognitive reappraisal in older adults. Each subtype of cognitive reappraisal was predicted to have a different influence on cognitive control abilities. Direct examination of the link between different cognitive reappraisals and specific aspects of cognitive control revealed that detached reappraisal relied heavily on cognitive control, whereas positive reappraisal did not. More importantly, mental set shifting was found to explain this disparity.

Prior studies of cognitive reappraisal largely focused on younger adults (John and Gross, 2004), leaving a knowledge gap in understanding of cognitive reappraisal processes used across lifespan. Identifying this gap is important, given that socioemotional selectivity theory asserts that emotion regulation becomes increasingly important with age (Carstensen et al., 1999).

To the best of our knowledge, this is the first study ever that examines how specific aspects of cognitive control, known as known as shifting, updating, and inhibition, might contribute to different subtypes of cognitive reappraisal. Subsequent results have implications for the creation and implementation of training interventions for older adults.

### Type Disparity in the Use of Cognitive Reappraisal

Employing both detached and positive reappraisals when faced with emotional stimuli is beneficial for one’s psychological well-being (Folkman, 1997; John and Gross, 2004; Shiota, 2006). As subtypes of cognitive reappraisal, both detached and positive reappraisal have been found to benefit psychological well-being when people are given emotionally charged stimuli (Folkman, 1997; John and Gross, 2004).

Previous studies have not addressed how detached and positive reappraisal take effects differently. This study examined detached and positive reappraisal usage in older adults observing while they were able to decrease subjective negative feelings by using either reappraisal method, their use of positive reappraisal was more effective; older adults displayed larger dampening of emotional reactions in positive reappraisal. Shiota and Levenson (2009) argued that as the use of positive reappraisal increased with age, older adults should be encouraged to use positive reappraisal more frequently to improve the quality of emotional life. However, the present study suggested that both reappraisal strategies are indispensable; indeed, both reappraisal strategies lead to reduced subjective negative feelings. More importantly, previous studies (Urry and Gross, 2010) showed that despite age-related differences in performance, people used both forms of reappraisal as they age. It is, therefore, important that individuals are able to utilize both strategies when facing highly emotional events.

An important question remains to be answered: what are the mechanisms that account for the deficient usage of detached reappraisal in older adults?

### Reliance on Cognitive Control of Detached Reappraisal

Shiota and Levenson (2009) attributed between-generational differences in detached reappraisal to different levels of cognitive control. Although their study provided inspiration for future studies, it did not provide a direct measure of its hypothesis and thus could not rule out other possible age-related factors. Opitz et al. (2012) conducted a neuroimaging study to examine the importance of cognitive control in detached reappraisal, observing reduced activation in dorsal medial prefrontal cortex and left ventrolateral prefrontal cortex in older adults. Such brain-related changes are believed to cause age-related declines in cognitive control, which gives rise to declines in the use of detached reappraisal. The present study showed no evidence that cognitive control impacted on detached reappraisal. However, the association was significant when looking into specific aspects of cognitive control.

Schmeichel et al. (2008) correlated working memory capacity with detached reappraisal, showing detached reappraisal relied on working memory capacity. However, working memory capacity cannot fully account for changes in cognitive control. Miyake and Friedman (2012) summarized a series of studies on cognitive control over the recent decades and developed the unity/diversity framework. Their framework proposed the system of cognitive control is made up of three elements: mental set shifting, information updating, and inhibition of prepotent responses. Furthermore each of these elements possesses both unity and diversity. Unity is inhibition of prepotent responses, an element itself, while diversity is element-specific. These three processes have shown to be correlated but remain distinct factors. Consistent with this framework, the present study confirmed the uniqueness of these three elements. Mental set shifting was the only element of cognitive control that correlated with detached reappraisal. As inhibition of prepotent responses, which is unity, had nothing to do with reappraisal effects, it was actually the shifting-specific part that played a role.

The present study did not find an association between age and the impact of detached reappraisal on subjective and physiological emotional reaction. This might be explained by the narrow age range of the study sample.

In the present study, subjective ratings of emotional experience were associated with HR, indicating an effect of detached reappraisal on both subjective and physiological participant reaction. Additionally, participants displayed reappraisal effects in skin conductance. Previous studies showed mixed results on physiological aspects of cognitive reappraisal. For example, Gross (1998) compared physiological reactions to disturbing film clips of participants assigned to either a cognitive reappraisal group or a control group; ultimately, no significant relationships was observed. Conversely, Urry (2010) reported significantly lower corrugator activity in a detached reappraisal condition compared to a just watch condition. However, for measures of HR and skin conductance (two measures used in the present study), Urry (2010) observed no associations between physiological measures and reappraisals of negative emotions. Skin conductance was also not a significant predictor of reappraisal effects in previous studies (Urry, 2010) despite the fact it is theoretically viewed as an objective measure of emotional reaction. Further work needs to be done to control unstable factors such as different skin conditions of participants that may affect quality of skin conductance data collection.

### Implications

This is the first study to identify a specific aspect of cognitive control (mental set shifting) to be associated with detached reappraisal. A direct examination of how different aspects of cognitive control influence cognitive reappraisal may help advance the understanding of cognitive reappraisal in older adults. Results of this study help establish specific aspects of cognitive control that might require training, providing important implications for the improvement of lives of older adults. For example, as mental set shifting was found to be responsible for the effects of detached reappraisal, training and improving the former may lead to higher quality emotional life in older adults.

Many studies (Baltes et al., 1998; Thompson and Foth, 2005) demonstrated cognitive plasticity in aging brains, which lead Thompson and Foth (2005) to conclude cognitive training intervention programs were effective for older adults. For example, a recent video game training study (Anguera et al., 2013) revealed robust plasticity of the prefrontal cognitive control system in older adults. That study focused on multitasking performance and showed transfer effects to other untrained cognitive control abilities. However, it must be acknowledged that such effective transfers to untrained cognitive control abilities are rather uncommon in literature, especially for older adults (Zelinski, 2009). The overwhelming majority of cognitive control training studies conducted focused on one aspect of cognitive control. It is likely observed failures in such training programs actually resulted from a failure of transfer, not training itself. That is, some training studies may not have found significant effects because they trained the wrong abilities and failed transfers; the training worked but there was no transfer. Results of the present study suggest it may be important to target specific aspects of cognitive control that could yield enhanced training effects in older adults.

## Author Contributions

All authors listed, have made substantial, direct and intellectual contribution to the work, and approved it for publication.

## Conflict of Interest Statement

The authors declare that the research was conducted in the absence of any commercial or financial relationships that could be construed as a potential conflict of interest. The reviewer NCS and handling Editor declared their shared affiliation, and the handling Editor states that the process nevertheless met the standards of a fair and objective review. The reviewer SH declared a shared affiliation, though no other collaboration, with one of the authors RK to the handling Editor, who ensured that the process nevertheless met the standards of a fair and objective review.
